# Postoperative chemotherapy had no prognostic effect on early‐staged young ovarian cancer with unilateral resection

**DOI:** 10.1002/cam4.1822

**Published:** 2018-10-10

**Authors:** Xiaofei Zhang, Shuoer Wang, SongJiao Zhao, Yidi Sun, Gong Yang

**Affiliations:** ^1^ Cancer Institute Fudan University Shanghai Cancer Center Shanghai China; ^2^ Department of Oncology, Shanghai Medical College Fudan University Shanghai China; ^3^ Central Laboratory The Fifth People's Hospital of Shanghai Fudan University Shanghai China; ^4^ Eye & ENT Hospital of Fudan University Shanghai China; ^5^ Key Lab of Computational Biology, CAS‐MPG Partner Institute for Computational Biology, Shanghai Institutes for Biological Sciences Chinese Academy of Sciences Shanghai China

**Keywords:** chemotherapy, ovarian cancer, premenopausal women, propensity score matching

## Abstract

Postoperative chemotherapy has been widely used in the treatment of early‐staged ovarian cancer patients underwent unilateral resection, but the clinical decision mainly depends on the doctor’s experience without a well‐defined guideline. This study used propensity score matching to analyze the effect of postoperative chemotherapy for early‐staged ovarian cancer patients underwent unilateral resection on prognosis. Patients of age 50 or younger than 50 with early‐staged ovarian cancer were explored from the Surveillance, Epidemiology, and End Results program database during 2000‐2018. Propensity score matching was used to randomize the dataset and reduce the selection biases. Univariate and multivariate cox proportional hazards models were utilized to estimate the necessity of chemotherapy. In univariate analysis of matched population, both the overall survival and cancer‐specific survival analysis showed that chemotherapy had no effect on the prognosis of early‐staged young ovarian cancer patients (Overall survival, *P = *0.477; Cancer‐specific survival, *P* = 0.950). In propensity‐adjusted multivariate analysis, chemotherapy still had no effect on both the overall and cancer‐specific survival probability after excluding the effect of all the confounding factors (HR = 0.863, CI = 0.587‐1.269, *P* = 0.455; HR = 1.009, CI = 0.633‐1.607, *P* = 0.970). Our study suggested that postoperative chemotherapy is not necessary for early‐staged young ovarian cancer patients with unilateral resection, as indicated by both the overall survival and cancer‐specific survival.

## 1 INTRODUCTION

Ovarian cancer, despite of the advances in detection and treatment, is still one of the most malignant tumors in female reproductive system with the highest mortality of 44.6% and the second highest morbidity of 20.9% according to the recent data.^1^. The peak age of the onset of ovarian cancer is over 50 years old, whereas the premenopausal patients under 50 years account for 38.5% of the whole. It is not well defined that whether chemotherapy should be used for early‐staged ovarian cancer patients undergoing unilateral resection in the guide NCCN 2016. The 5‐year overall and cancer‐specific survival rate were both over 90% based on previous studies. Postoperative chemotherapy has great impact on the quality of life and fertility, especially the young patients who have baby needs. Therefore, it is important to avoid unnecessary chemotherapy. Up to now, whether to undergo postoperative chemotherapy for early‐staged ovarian cancer patients is mainly determined by physician’s experience, which may lead to selection bias.

This study used propensity score matching to analyze the effect of postoperative chemotherapy for early‐staged ovarian cancer patients undergoing unilateral resection on survival rate, which can provide a reference for physicians to reduce the possibility of selection bias.

## 2 MATERIAL AND METHODS

### 2.1 Clinical dataset

We included young patients (<50 years old) with early‐staged ovarian cancers diagnosed between 2000 and 2014 from 18 population‐based registries of the Surveillance, Epidemiology, and End Results (SEER), the largest cancer database in the United States, in our study. The SEER database was downloaded from the official website (https://seer.cancer.gov/about/overview.html). Patients without available surgical method or chemotherapy information were excluded from our analysis. Primary ovarian cancer was identified with code "C56.9" according to the *International classification of Diseases for Oncology*, Third Edition (ICD‐O‐3), and unilateral resection of ovarian was selected by SEER surgical code manual. Chemotherapy information was retrieved individually after getting approval from the SEER official. Besides, we also considered age at diagnosis, marital status, race, tumor grade, American Joint Committee on Cancer (AJCC) stages, tumor size, registry, lateral of original tumor, and histological type for each patient. Cases with no complete survival information including vital status, cause of death, and survival time were removed from further study. We grouped patients into <30 years old, 30‐40 years, and more than 40 years old. Race was classified into four groups of American Indian/Alaska Native (AI/AN), Asian, Black, and White. Tumor grade was classified into well differentiated (G1), moderately differentiated (G2), poorly differentiated (G3), and undifferentiated (G4). We only considered patients with IA, IB, IC, or IIA ovarian cancers based on the criteria of American Joint Committee on Cancer (AJCC) Staging Manual, 7th edition, 2010. Tumor size was divided into three categories by cutoffs of 2 and 10 cm. The 18 registries were grouped into three classes according to the geographical location, central (Metropolitan Detroit, Iowa, Kentucky, Utah, and Louisiana), east (New Jersey, Metropolitan Atlanta, Rural Georgia, and Greater Georgia), and west (Alaska, Greater California, Hawaii, Los Angeles, New Mexico, San Francisco‐Oakland SMSA, San Jose‐Monterey, and Seattle). All the patients were grouped into three histological types epithelial, germ‐cell tumor, and sex‐cord‐stromal tumor according to the ICD‐O‐3 SEER site‐specific manual, and the final analytic set consisted of 1849 cases of patients thereafter.

### 2.2 Propensity score matching

Propensity score matching was used in the study to avoid the influence of selection bias to the conclusion. Selection bias was generally existed in retrospective studies because of the heterogeneity of demographic and clinical characteristics between the treatment group and control group. A multivariate logistic model was fit by clinical factors including age at diagnosis, marital status, race, tumor grade, tumor stage, tumor size, registry, lateral and tumor histology to predict the probability of a patient receiving chemotherapy. The propensity score was ranged from 0 to 1, and patients with similar propensity scores from the treatment group and control group were matched until all patients in the group with smaller size patients got a match. The nearest neighbor algorithm and one by one match approach were applied in the model, and R package "MatchIT" was used for this analysis.

### 2.3 Survival analysis

The survival curves were generated by Kaplan‐Meier in the study, and log‐rank test was applied to calculate differences between the curves. Univariate and multivariate cox proportional hazards models were applied for estimating hazard ratios (HRs) and 95% confidence intervals (CI) for each variate by the R package “survival”.

### 2.4 Statistical analysis

All the statistical analyses in this work were conducted with R version 3.3.2 (https://www.R-project.org/). The differences of clinicopathological characteristics with or without chemotherapy were analyzed by chi‐square (*χ* ^2^) test both before and after the propensity score matching. All tests conducted were two‐sided, and significant difference was considered as *P*‐value <0.05.

## 3 RESULTS

### 3.1 Clinical characteristics of the study cohort

We included 1849 early‐staged young ovarian patients who underwent unilateral resection surgery in the study, and 71.9% of the cohort also had no chemotherapy in addition to the surgery. Among the whole population, nearly 50% of the cases were diagnosed at age <30%, and 60.6% of the cohort were single women. White patients constituted 74.2% of the cohort, while the remaining were composed of Asian, Black, and American Indians/Alaska native. Only 52% of patients were available with information of tumor grade, and most of them were well or moderately differentiated (18.8% and 16.8%, respectively). Stage IA and IC patients constituted almost the whole set, with relative 77.5% and 20.4% of the population. For the patients with known tumor sizes, 38.9% of patients had tumors with diameter more than 10 cm. More than half of the study cohort were from western registries, and the primary tumor was equally located on the left or right side of the ovarian. Epithelial ovarian cancer was the most popular in the dataset with the proportion of 55.9%, and sex‐cord‐stromal tumor only made up for 9.1% (Table 1).

**Table 1:** Demographic and clinical characteristics of patients with ovarian cancer

Characteristics	Number	Percentage (%)
Chemotherapy (%)		
Chemotherapy−	1330	71.9
Chemotherapy+	519	28.1
Age (%)		
<30 y	908	49.1
30‐40 y	450	24.3
>40 y	491	26.6
Marital status (%)		
Married	650	35.2
Single	1121	60.6
Unknown	78	4.2
Race (%)		
AI/AN	13	0.7
Asian	229	12.4
Black	209	11.3
White	1372	74.2
Unknown	26	1.4
Grade (%)		
Well differentiated	347	18.8
Moderately differentiated	310	16.8
Poorly differentiated	223	12.1
Undifferentiated	81	4.4
Unknown	888	48
Stage (%)		
IA	1433	77.5
IB	7	0.4
IC	378	20.4
IIA	31	1.7
Tumor Size (%)		
<2 cm	142	7.7
2‐10 cm	445	24.1
>10 cm	719	38.9
Unknown	543	29.4
Registry (%)		
Central	328	17.7
East	466	25.2
West	1055	57.1
Lateral (%)		
Left	940	50.8
Right	889	48.1
Unknown	20	1.1
Histology (%)		
Epithelial	1033	55.9
Germ‐cell tumor	647	35
Sex‐cord‐stromal tumor	169	9.1

### 3.2 Comparison of covariates before and after propensity score matching

Before the propensity score matching, patients undergoing chemotherapy tended to be diagnosed at younger ages (56.5% vs 46.2% with age <30 years, *P* < 0.001) and unmarried (32.8% vs 36.1%, *P* = 0.016). They were less differentiated (7.7% vs 23.1% with well‐differentiated tumor, *P* < 0.001), less likely to be in stage IA (59.9% vs 84.4%), larger tumor sizes (51.8% vs 33.8% with tumor size more than 10 cm, *P* < 0.001), and larger proportion of germ‐cell tumors (50.9% vs 28.8%, *P* < 0.001). Propensity score matching was then performed on the initial dataset to eliminate the heterogeneity and imbalance between the group with or without chemotherapy by building a regression model integrating age at diagnosis, marital status, tumor grade, tumor stage, tumor size and histology. Actually, after the propensity score matching, the two groups with or without chemotherapy were equal in the number of patients, and the clinical factors were well balanced without significant differences, indicating the potential covariates between groups were greatly decreased (Table 2).

**Table 2:** Clinical characteristics of the study cohort before and after propensity score matching

Characteristics	Before Propensity score matching			After Propensity score matching		
	Chemotherapy− (n = 1330)	Chemotherapy+ (n = 519)	*P*‐value	Chemotherapy− (n = 1330)	Chemotherapy+ (n = 519)	*P*‐value
Age (%)						
<30 y	615 (46.2)	293 (56.5)	<0.001	307 (59.2)	293 (56.5)	0.588
30‐40 y	344 (25.9)	106 (20.4)		94 (18.1)	106 (20.4)	
>40 y	371 (27.9)	120 (23.1)		118 (22.7)	120 (23.1)	
Marital status (%)						
Married	480 (36.1)	170 (32.8)	0.016	154 (29.7)	170 (32.8)	0.561
Single	785 (59.0)	336 (64.7)		351 (67.6)	336 (64.7)	
Unknown	65 (4.9)	13 (2.5)		14 (2.7)	13 (2.5)	
Race (%)						
American Indian	8 (0.6)	5 (1.0)	0.36	2 (0.4)	5 (1.0)	0.634
Asian	160 (12.0)	69 (13.3)		70 (13.5)	69 (13.3)	
Black	142 (10.7)	67 (12.9)		59 (11.4)	67 (12.9)	
White	999 (75.1)	373 (71.9)		380 (73.2)	373 (71.9)	
Unknown	21 (1.6)	5 (1.0)		8 (1.5)	5 (1.0)	
Grade (%)						
Well differentiated	307 (23.1)	40 (7.7)	<0.001	44 (8.5)	40 (7.7)	0.071
Moderately differentiated	195 (14.7)	115 (22.2)		131 (25.2)	115 (22.2)	
Poorly differentiated	105 (7.9)	118 (22.7)		98 (18.9)	118 (22.7)	
Undifferentiated	33 (2.5)	48 (9.2)		29 (5.6)	48 (9.2)	
Unknown	690 (51.9)	198 (38.2)		217 (41.8)	198 (38.2)	
Stage (%)						
IA	1122 (84.4)	311 (59.9)	<0.001	353 (68.0)	311 (59.9)	0.045
IB	6 (0.5)	1 (0.2)		1 (0.2)	1 (0.2)	
IC	183 (13.8)	195 (37.6)		152 (29.3)	195 (37.6)	
IIA	19 (1.4)	12 (2.3)		13 (2.5)	12 (2.3)	
Tumor size (%)						
<2 cm	127 (9.5)	15 (2.9)	<0.001	11 (2.1)	15 (2.9)	0.601
2‐10 cm	318 (23.9)	127 (24.5)		119 (22.9)	127 (24.5)	
>10 cm	450 (33.8)	269 (51.8)		266 (51.3)	269 (51.8)	
Unknown	435 (32.7)	108 (20.8)		123 (23.7)	108 (20.8)	
Registry (%)						
Central	225 (16.9)	103 (19.8)	0.12	82 (15.8)	103 (19.8)	0.191
East	327 (24.6)	139 (26.8)		137 (26.4)	139 (26.8)	
West	778 (58.5)	277 (53.4)		300 (57.8)	277 (53.4)	
Lateral (%)						
Left	682 (51.3)	258 (49.7)	0.776	280 (53.9)	258 (49.7)	0.385
Right	633 (47.6)	256 (49.3)		235 (45.3)	256 (49.3)	
Unknown	15 (1.1)	5 (1.0)		4 (0.8)	5 (1.0)	
Histology (%)						
Epithelial	811 (61.0)	222 (42.8)	<0.001	221 (42.6)	222 (42.8)	0.479
Germ‐cell tumor	383 (28.8)	264 (50.9)		255 (49.1)	264 (50.9)	
Sex‐cord‐stromal tumor	136 (10.2)	33 (6.4)		43 (8.3)	33 (6.4)	

### 3.3 Chemotherapy has no significant benefit on the survival of early‐staged ovarian cancer patients undergoing unilateral resection

**Figure 1:**
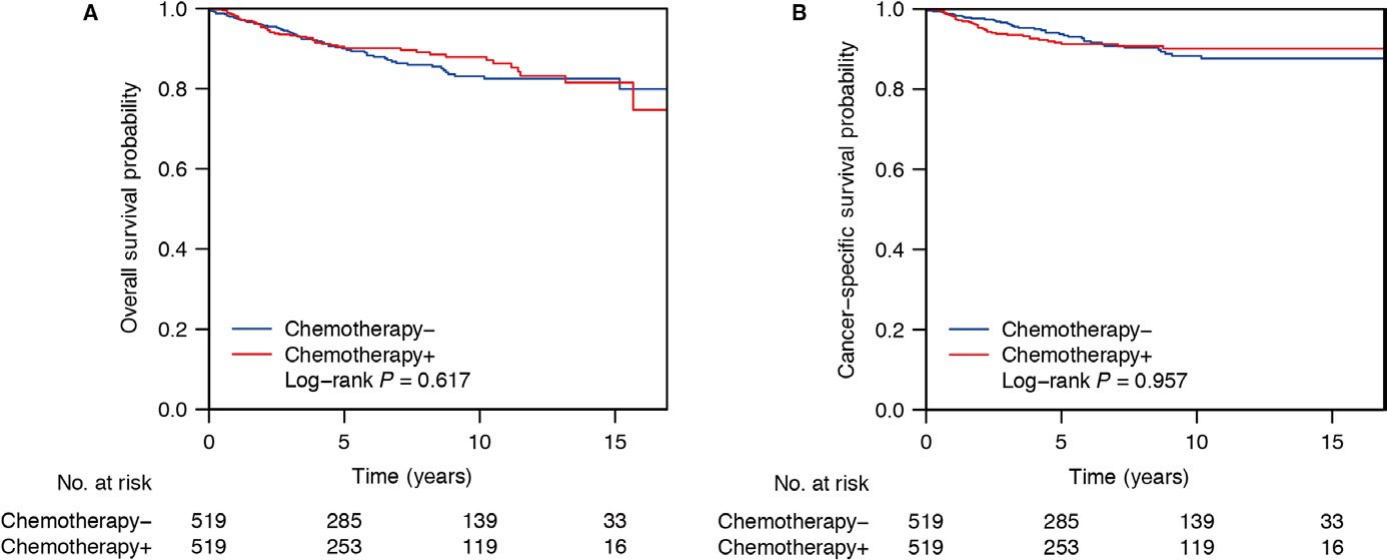
Chemotherapy demonstrated no significant benefit to patients with early‐staged ovarian cancer who underwent unilateral resection for both overall survival and cancer‐specific survival. Overall survival probability (A) or cancer‐specific survival probability (B) of patients with early‐staged ovarian cancer who underwent unilateral resection underwent or without chemotherapy

Chemotherapy demonstrated no significant benefit to patients with early‐staged ovarian cancer who underwent unilateral resection for both overall survival and cancer‐specific survival (Overall survival, *P* = 0.396; Cancer‐specific survival, *P* = 0.996; Figure 1). For all the stages included in this study, patients with chemotherapy did not show any survival differences compared with those underwent no chemotherapy (Table 3 and Supplementary Figure S1).

**Table 3:** Five year survival probability in different stages of ovarian cancer

	Overall survival			Cancer‐specific survival		
	Chemotherapy−	Chemotherapy+	*P*‐value	Chemotherapy−	Chemotherapy+	*P*‐value
IA	0.918 (0.017)	0.933 (0.016)	0.396	0.947 (0.014)	0.941 (0.015)	0.996
IB	1 (0)	1 (0)	1	1 (0)	1 (0)	1
IC	0.861 (0.033)	0.854 (0.03)	0.864	0.905 (0.029)	0.871 (0.029)	0.902
IIA	0.666 (0.138)	0.771 (0.144)	0.491	0.666 (0.138)	0.771 (0.144)	0.491

### 3.4 Univariate and multivariate analysis

We conducted univariate cox proportional hazard analyses on the matched population of all the clinical characteristics to explore their prognostic effect (Table 4). Both the overall survival and cancer‐specific survival analysis showed that chemotherapy had no effect on the prognosis of early‐staged young ovarian cancer patients (Overall survival, *P = *0.477; Cancer‐specific survival, *P* = 0.950). Older ages demonstrated a hazard factor for both the overall and cancer‐specific survival (*P* < 0.001), while race only showed overall survival differences among groups (*P* = 0.007). Both tumor grade and stage were significantly associated with overall and cancer‐specific survival (*P* < 0.001), and tumor size was only correlated with cancer‐specific survival probability (*P* = 0.042). Besides, histology demonstrated a significant risk factor for both the overall and survival rate (*P* < 0.001). Further, we took all the risk factors associated with survival from the univariate analysis into a multivariate cox proportional model with chemotherapy, and the result was shown in Table 5. Age more than 40 years remained a risk factor in the overall survival but not for the cancer‐specific survival (HR = 2.372, CI = 1.329‐4.233, *P* = 0.003; HR = 1.261, CI = 0.672‐2.365, *P* = 0.471). Stage IC was associated with poorer survival probabilities than stage IA patients (HR = 1.753, CI = 1.178‐2.609, *P* = 0.006; HR = 2.212, CI = 1.357‐3.604, *P* = 0.001), while stage IIA only correlated with worse cancer‐specific survival (HR = 3.502, CI = 1.426‐8.599, *P = *0.006). Poorly differentiated (HR = 5.801, CI = 1.769‐19.010, *P* = 0.004; HR = 5.089, CI = 1.520‐17.030, *P* = 0.008) and undifferentiated tumors (HR = 5.768, CI = 1.604‐20.730, *P* = 0.007; HR = 5.107, CI = 1.356‐19.230, *P* = 0.016) were both associated with worse survival compared to the well‐differentiated tumor. Chemotherapy still had no effect on both the overall and cancer‐specific survival probability after excluding the effect of all the confounding factors (HR = 0.863, CI = 0.587‐1.269, *P* = 0.455; HR = 1.009, CI = 0.633‐1.607, *P* = 0.970).

**Table 4:** Univariate analysis of the matched population for overall and cancer‐specific survival

Characteristics	N	Overall		Cancer‐specific	
		5‐year survival (%)	*P*‐value	5‐year survival (%)	*P*‐value
Chemotherapy (%)					
Chemotherapy−	519	0.895	0.477	0.928	0.950
Chemotherapy+	519	0.901		0.912	
Age (%)					
<30 y	600	0.960	<0.001	0.967	<0.001
30‐40 y	200	0.907		0.922	
>40 y	238	0.752		0.811	
Marital Status (%)					
Married	324	0.892	0.835	0.903	0.612
Single	687	0.902		0.929	
Unknown	27	0.882		0.941	
Race (%)					
American Indian	7	1.000	0.007	1.000	0.106
Asian	139	0.966		0.966	
Black	126	0.899		0.912	
White	13	1.000		1.000	
Unknown	753	0.885		0.912	
Grade (%)					
Well differentiated	84	0.953	<0.001	0.953	<0.001
Moderately differentiated	246	0.906		0.932	
Poorly differentiated	216	0.820		0.859	
Undifferentiated	77	0.840		0.851	
Unknown	415	0.935		0.951	
Stage (%)					
IA	664	0.925	<0.001	0.944	<0.001
IB	2	1.000		1.000	
IC	347	0.856		0.885	
IIA	25	0.718		0.718	
Tumor Size (%)					
<2 cm	26	0.800	0.067	0.800	0.042
2‐10 cm	246	0.874		0.896	
>10 cm	535	0.918		0.937	
Unknown	231	0.889		0.920	
Registry (%)					
Central	185	0.881	0.708	0.919	0.976
East	276	0.895		0.917	
West	577	0.905		0.922	
Lateral (%)					
Left	538	0.903	0.188	0.929	0.048
Right	491	0.899		0.918	
Unknown	9	0.571		0.571	
Histology (%)					
Epithelial	443	0.808	<0.001	0.840	<0.001
Germ‐cell tumor	519	0.987		0.998	
Sex‐cord‐stromal tumor	76	0.833		0.865	

**Table 5:** Multivariate cox proportional model of the matched population for overall and cancer‐specific survival

	Overall			Cancer‐specific		
	HR	95% CI	*P*‐value	HR	95% CI	*P*‐value
Age (%)						
<30 y	Ref			Ref		
30‐40 y	1.193	0.613‐2.319	0.603	1.074	0.5252.197	0.846
>40 y	2.372	1.329‐4.233	0.003	1.261	0.672‐2.365	0.471
Race (%)						
American Indian	Ref			Ref		
Asian	2813953.873	0‐Inf	0.995	4505754.487	0‐Inf	0.996
Black	7987742.825	0‐Inf	0.995	12587762.630	0‐Inf	0.996
White	6835996.312	0‐Inf	0.995	7511476.490	0‐Inf	0.996
Unknown	1.139	0‐Inf	1.000	1.440	0‐Inf	1.000
Stage (%)						
IA	Ref			Ref		
IB	0.000	0‐Inf	0.999	0.000	0‐Inf	0.999
IC	1.753	1.178‐2.609	0.006	2.212	1.357‐3.604	0.001
IIA	1.926	0.812‐4.562	0.136	3.502	1.426‐8.599	0.006
Grade (%)						
Well differentiated	Ref			Ref		
Moderately differentiated	3.092	0.925‐10.33	0.067	2.287	0.662‐7.903	0.191
Poorly differentiated	5.801	1.769‐19.01	0.004	5.089	1.520‐17.03	0.008
Undifferentiated	5.768	1.604‐20.73	0.007	5.107	1.356‐19.23	0.016
Unknown	3.311	0.997‐10.99	0.051	2.277	0.655‐7.917	0.196
Histology (%)						
Epithelial	Ref			Ref		
Germ‐cell Tumor	0.161	0.072‐0.360	<0.001	0.017	0.002‐0.126	<0.001
Sex‐cord‐stromal Tumor	0.910	0.455‐1.818	0.790	0.736	0.333‐1.623	0.447
Chemotherapy (%)						
Chemotherapy−	Ref			Ref		
Chemotherapy+	0.863	0.587‐1.269	0.455	1.009	0.633‐1.607	0.970

## 4 DISCUSSION

We conducted a population‐based study on young women with early‐staged ovarian cancer to explore the necessity of postoperative chemotherapy for such patients who undergo unilateral resection. The propensity score matching was used to help randomize the dataset and strengthen causal arguments by reducing selection bias of diagnosis. The univariate and multivariate cox proportional hazard model analysis suggested that postoperative chemotherapy is not necessary for young patients undergoing unilateral resection therapy.

For young women of childbearing age, the reproductive ability has to be taken into account. Combination therapy of postoperative patients with cisplatin and paclitaxel is recommended by the NCCN guide.^2^ Cisplatin directly binds to the DNA of tumor cells, forming a cross‐link that leads to the arrest of DNA synthesis and replication, resulting in apoptosis.^3^ Paclitaxel mainly blocks cancer growth by binding to the tubulin proteins needed for cell division.^4^ However, both of the drugs may cause several types of side effects due to the lack of selectivity and the cytotoxicity of the target. One of the major side effects that cannot be neglected is the reproductive toxicity due to the disease’s sex selection. For pregnant women, chemotherapy with cisplatin and paclitaxel during the second and third trimesters with these two drugs may lead to a relatively high risk of premature rupture of membranes (PROM), intrauterine growth restriction (IUGR), and premature labor,^5^, ^6^ according to available data. Besides, first trimester chemotherapy exposure is associated with fetal malformations, spontaneous abortions, and fetal death.^7^ In clinical practice, women undergoing chemotherapy should avoid pregnancy, and termination should be considered in patients with cancer who need systemic treatment in the first trimester. Therefore, the exemption of postoperative chemotherapy means a lot to the early‐staged young women with ovarian preservation and willing to have a child.

Aside from the concerns for reproductive ability, there are some other side effects in the use of cisplatin and paclitaxel, such as bone marrow suppression, which may lead to anemia, infection, and fever.^8^ Neurotoxicity, which damages motor and sensory nerves.^9^, ^10^ Nausea, vomiting, and dermatitis are also common symptoms after using these two drugs.^11^ In addition, the nephrotoxicity of cisplatin may lead to acute kidney injury (AKI) or irreversible renal dysfunction.^12^ Hence, the conduction of postoperative chemotherapy should be cautiously considered by the operatives.

Even though we included large number of cases and applied bias reduction method to ensure the accuracy and reliability of our study, there are still some concerns that need to be pointed out. Firstly, we didn’t consider the individual difference and assuming women younger than 50 years old were premenopausal. Secondly, as the information of specific drugs in chemotherapy was not available in the database, we only considered the influence of whether applying the therapy or not. Inclusion of chemotherapy dose and duration is preferred in further analysis to give more precise conclusion. Besides, despite that we considered as many as potential clinical cofactors in our analysis, the limited information on surgical and treatment options such as the procedure strategy, specimen adequacy, and the judgement of pathologists was still overlooked for their influence on prognosis.

In summary, our study suggested that postoperative chemotherapy is not necessary for early‐staged young ovarian cancer patients with unilateral resection, as indicated by both the overall survival and cancer‐specific survival. The exemption of postoperative chemotherapy will do great benefit to young women with childbearing ability and wishes without reducing the curative effects. Nevertheless, a comprehensive risk assessment from the physicians and associated tests is strongly recommended. In addition, randomized clinical trials are needed to further evaluate the necessity of postoperative chemotherapy for targeted patients.

## CONFLICT OF INTEREST

The authors declare no conflict of interests.
