# Supplementary figures and images for: Postoperative chemotherapy had no prognostic effect on early‐staged young ovarian cancer with unilateral resection
Source: Cancer Med. 2018 Oct 10;7(11):5488–96. doi: 10.1002/cam4.1822 (PMC6246923; doi:10.1002/cam4.1822)

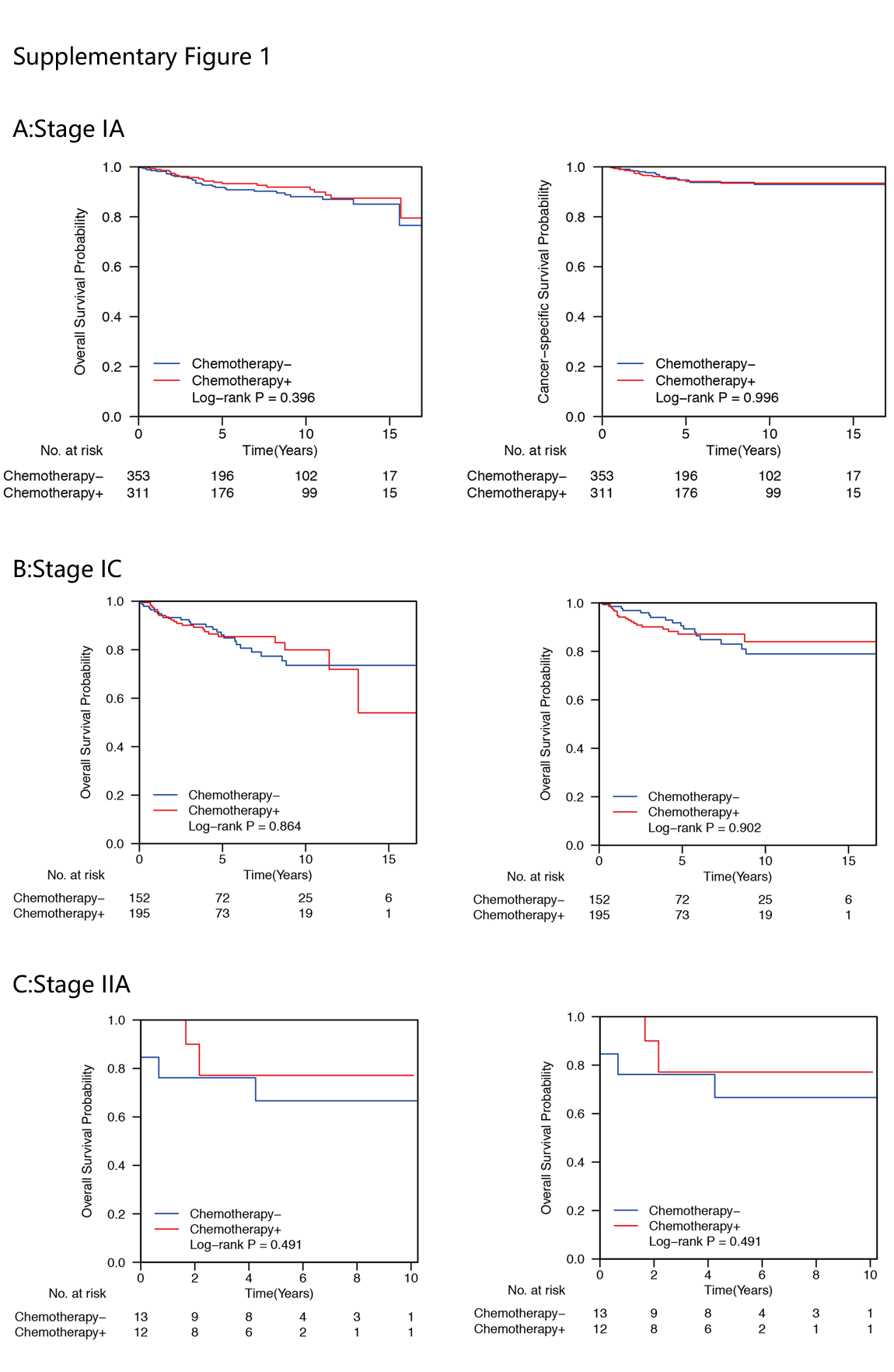

Supplement: Supplementary file 1 [file CAM4-7-5488-s001.tif]
